# Impaired Proliferation of CD8^+^ T Cells Stimulated with Monocyte-Derived Dendritic Cells Previously Matured with Thapsigargin-Stimulated LAD2 Human Mast Cells

**DOI:** 10.1155/2024/5537948

**Published:** 2024-07-18

**Authors:** Katerina Kalkusova, Pavla Taborska, Dmitry Stakheev, Michal Rataj, Sindija Smite, Elea Darras, Julia Albo, Jirina Bartunkova, Luca Vannucci, Daniel Smrz

**Affiliations:** ^1^ Department of Immunology Second Faculty of Medicine Charles University and University Hospital Motol, Prague, Czech Republic; ^2^ Laboratory of Immunotherapy Institute of Microbiology of the Czech Academy of Sciences, Prague, Czech Republic

## Abstract

CD8^+^ T cells are essential for adaptive immunity against infection and tumors. Their ability to proliferate after stimulation is crucial to their functionality. Dendritic cells (DCs) are professional antigen-presenting cells that induce their proliferation. Here, we show that thapsigargin-induced LAD2 mast cell (MC) line-released products can impair the ability of monocyte-derived DCs to induce CD8^+^ T-cell proliferation and the generation of Th1 cytokine-producing T cells. We found that culture medium conditioned with LAD2 MCs previously stimulated with thapsigargin (thapsLAD2) induces maturation of DCs as determined by the maturation markers CD80, CD83, CD86, and HLA-DR. However, thapsLAD2-matured DCs produced no detectable TNF*α* or IL-12 during the maturation. In addition, although their surface expression of PD-L1 was comparable with the immature or TLR7/8-agonist (R848)-matured DCs, their TIM-3 expression was significantly higher than in immature DCs and even much higher than in R848-matured DCs. In addition, contrary to R848-matured DCs, the thapsLAD2-matured DCs only tended to induce enhanced proliferation of CD4^+^ T cells than immature DCs. For CD8^+^ T cells, this tendency was not even detected because thapsLAD2-matured and immature DCs comparably induced their proliferation, which contrasted with the significantly enhanced proliferation induced by R848-matured DCs. Furthermore, these differences were comparably recapitulated in the ability of the tested DCs to induce IFN*γ*- and IFN*γ*/TNF*α*-producing T cells. These findings show a novel mechanism of MC-mediated regulation of adaptive immune responses.

## 1. Introduction

Mast cells (MCs) are long-lived terminally differentiated tissue or mucosa-resident cells [1]. Although these cells are largely associated with asthma, allergy, atopy, and anaphylaxis [2, 3], they also play a significant role in cancer development, autoimmune diseases, and regulation of other components of the immune system under physiologic or pathologic conditions [4, 5, 6]. The regulation can be mediated either via direct contact with other immune cells [7, 8] or through biologically active products released after their stimulation [9, 10]. One of the immune cells that can be affected by MCs are dendritic cells (DCs) [7, 11, 12, 13, 14].

DCs are professional antigen-presenting cells responsible for the induction of adaptive immune responses [15]. These cells first differentiate from hematopoietic progenitors into immature DCs [16]. Immature DCs have high phagocytic capacity and express low levels of costimulatory molecules such as CD80, CD86, CD83, and MHC II and secrete no or low levels of immunostimulatory or immunoregulatory cytokines, such as TNF-*α*, IL-12, or IL-10 [15, 17, 18]. The cytokine IL-12 is an essential signaling molecule driving the immune response toward the type 1 T helper (Th1) response and is often known as a third signal for CD8^+^ T-cell proliferation [19]. Immature DCs also express pattern recognition receptors (PRRs) [20], including Toll-like receptors (TLRs) [21]. These receptors recognize pathogen-associated molecular patterns (PAMPs) [20] or damage-associated molecular patterns (DAMPs) [22] molecules. PRR triggering by PAMPs or DAMPs is interpreted by immature DCs as danger signals that activate them and lead to their maturation [23]. Matured DCs are phenotypically changed. They already express high levels of costimulatory molecules and immunostimulatory cytokines [23]. This allows matured DCs to induce adaptive immune responses through effective induction of T-cell proliferation and differentiation [24]. The ability of matured DCs is essential for effective and sustained adaptive immunity. However, this ability is often compromised under disease conditions, such as in the tumor microenvironment, where multiple infiltrating immune cells can impact the DC functionality.

One type of tumor-infiltrating immune cell is MCs. The functionality of DCs can be impacted by MCs through either direct interaction or MC-released products [9, 10]. The direct interaction can mediate cell-to-cell crosstalk through the creation of synapses, which can, for instance, transfer antigens from MCs to DCs [7]. The MCs released products contain multiple biologically active products and can modulate DC functionality and migration [9]. These products are released from activated MCs following different stimuli. One of these stimuli is thapsigargin, which is an irreversible inhibitor of sarco/endoplasmic reticulum Ca2^+^-ATPases (SERCA) [25, 26, 27]. These ATPases pump leaked calcium back to the endoplasmic reticulum [28], and their inhibition by thapsigargin is sufficient to trigger intracellular calcium mobilization necessary for the activation of calcium signaling [29]. The activated calcium signaling in MCs leads to their activation and subsequent anaphylactic degranulation [30]. We have previously investigated whether a well-defined human MC line, LAD2 [31], could be used as a cellular adjuvant for the maturation of *ex vivo*-produced monocyte-derived DCs [32]. We found that the coculture of immature DCs with thapsigargin-stimulated LAD2 MCs induces DC maturation that can be attained even with a maturation-refractory phenotype of immature DCs [32]. In the present study, we investigated whether this LAD2 MC-mediated DC maturation is contact-dependent and what is the functionality of such matured DCs as compared with their immature or TLR7/8 agonist-matured counterparts.

## 2. Materials and Methods

### 2.1. Specimens

LAD2 MCs were obtained under MTA (NIAID no. 2021-0616) from NIAID/NIH [31]. The buffy coats from healthy donors were from the Institute of Hematology and Blood Transfusion in Prague, and the donors signed written informed consent to participate in the study. The institutional research committee approved all experimental protocols—the Ethics Committee of the University Hospital Motol in Prague (reference number: EK-753.1.8/21). The experiments were performed in accordance with the 1964 Helsinki Declaration and its later amendments or comparable ethical standards.

### 2.2. Preparation of Monocyte-Derived Immature DCs

Peripheral blood mononuclear cells (PBMCs) were isolated from buffy coats of healthy donors by density gradient separation and cryopreserved as described previously [33, 34]. The cryopreserved PBMCs were reconstituted, fractioned by plastic adherence into lymphocytes (nonadherent fraction) and monocytes (adherent fraction), and the monocytes were differentiated into immature DCs (iDCs) in a serum-containing culture medium [KM medium; RPMI 1640 medium (Thermo Scientific, Waltham, MA, USA), 10% heat-inactivated fetal bovine serum (HyClone, GE Healthcare Life Sciences, South Logan, UT, USA), 100 U/ml penicillin-streptomycin, 2 mM GlutaMax (Thermo Scientific)] supplemented with 1,000 IU/ml of GM-CSF and 1,000 IU/ml of IL-4 (Immunotools, Friesoythe, Germany) as described previously [33, 34, 35]. The produced iDCs in the cell culture were then directly used for further analyses.

### 2.3. LAD2 MC Stimulation and Medium Conditioning

LAD2 MCs were cultured in a serum-free culture medium as described previously [32]. The cells (0.5–1.0 × 10^6^ cells/ml) were harvested, two-times rinsed with 5 ml DC culture medium, and stimulated with thapsigargin (0.5 *µ*M; Sigma-Aldrich, St. Louis, MO, USA) [36, 37] for 30 min at 37°C and 5% CO_2_. The cells stimulated with the vehicle alone were used as control (unstimulated) cells. The thapsigargin- or vehicle-stimulated cells were then extensively rinsed three times with 10–25 ml of KM medium. The cells were then resuspended in KM medium at the concentration of 2.0 × 10^6^ cells/ml, and 1 ml of the suspension was transferred to F-bottom 48-well plate wells. The cells were then cultured for 18–24 hr at 37°C and 5% CO_2_. The supernatant of the cultured cells (800 *µ*l) was gently recovered from the top of undisturbed wells and pooled in a 15-ml centrifuge tube. The pooled supernatant was centrifuged at room temperature (300× *g*, 8 min). To avoid contamination of the supernatant with the pelleted material, the supernatant was gently recovered from the top of the undisturbed tube with repeated use of a 1 ml pipette until approximately 300 *µ*l of the volume remained at the bottom of the tube. The recovered supernatant was gently stirred, aliquoted, and stored at −20°C.

### 2.4. DC Maturation

The produced iDCs in the cell culture were harvested and pelleted at 240 × *g* for 10 min at room temperature. The pelleted cells were resuspended in KM supplemented with GM-CSF and IL-4 (1,000 IU/ml; Immunotools) at a concentration of 2 × 10^6^ cells/ml. The cell suspension (100 *µ*l; 0.2 × 10^6^ iDCs) was transferred to U-bottom 96-well plate wells with 100 *µ*l of the cytokine-supplemented KM medium combined, or not, with the conditioned supernatant at indicated dilutions. As a positive control, the cells were supplemented with R848 (10 *µ*g/ml; Enzo Life Sciences, Farmingdale, NY, USA). The cells were extensively resuspended and cultured for 18–24 hr (37°C, 5% CO_2_). For the determination of the cytokine production during the maturation, iDCs were additionally supplemented with brefeldin A (BioLegend, San Diego, CA, USA) after 1 hr poststimulation of DC maturation.

### 2.5. DC Maturation Analysis

For the determination of DC maturation, the cells were transferred to a V-bottom 96-well plate (Nalgene, Rochester, NY, USA) and extracellular molecules were stained as described previously [34] with the following antibodies: CD11c-APC (Exbio, Prague, Czech Republic) with either CD80-FITC, CD86-PE, CD83-FITC (Beckman Coulter, Brea, CA, USA), HLA-DR-Pe-Cy7 (Becton Dickinson), or TIM-3-PE (BioLegend, San Diego, CA, USA) and PD-L1 (CD274)-APC (eBiosciences, San Diego, CA, USA). The stained cells were analyzed by FACSAria II, BD LSR Fortessa flow cytometers (Becton Dickinson), or NovoCyte Advanteon (Agilent, Santa Clara, CA, USA). The acquired data were processed with the FlowJo software (Tree Star, Ashland, OR, USA). The extent of viable cells was determined by the frequency of the DAPI-negative cell population. The proportions of DCs were determined as the frequency of the CD11c-positive cell population. The extent of the DC maturation was determined as the mean fluorescence intensity staining for each maturation marker.

For the determination of the cytokine production during the maturation, DCs were stained (TNF*α* staining) or not (IL-12 staining), with a fixable live/dead stain (Vivid, Thermo Scientific), then fixed and permeabilized as previously described [38]. For TNF*α* staining, the Vivid-stained cells were then stained with TNF*α*-APC (Becton Dickinson, Franklin Lakes, NJ, USA). For IL-12 staining, the cells were stained with CD11c-APC (Exbio) and IL-12-BV421 (Becton Dickinson). For TNF*α* staining, the extent of viable cells was determined as the frequency of the Vivid negative cell population. Since the CD11c-specific antibody had the same fluorochrome as the TNF*α*-specific antibody (APC), the CD11c antibody was not used in the panel, and the DC population was gated using a surrogate SSC/FSC gating strategy, which was shown to correspond with the CD11c-positive (DC) population in the IL-12 staining panel. For IL-12 staining, the extent of viable cells was determined as the frequency of the monocyte-like population, which corresponded with the CD11c population (DCs). The frequency of the cytokine-producing DCs was determined for individual cytokine staining of the DC populations. The IL-12 released into the supernatant was determined with Duo-Set ELISA (R&D Systems, Minneapolis, MN, USA). The amount of IL-12 was expressed as the concentration (pg/ml) of IL-12 released by 1 × 10^6^ cells/ml.

### 2.6. Ki-67 and CFSE Proliferation Assays, TIM-3, and PD-1 Staining

Maturated or immature DCs were gamma irradiated (32 Gy) using Gammacell 3000 ELAN (Best Theratronics, Ottawa, ON, Canada). The irradiated DCs were combined with allogeneiclymphocytes (nonadherent fraction of PBMCs) at a 1 : 10 ratio (DCs: lymphocytes) in a U-bottom 96-well plate and cocultured in human plasma serum-containing culture medium [RPMI 1640 medium, 5% human plasma serum (One Lambda, Canoga Park, CA, USA), 100 U/ml penicillin-streptomycin, 2 mM GlutaMax, 1 mM sodium pyruvate, and 1 mM nonessential amino acid mix (Thermo Scientific)] for 5 days (37°C, 5% CO_2_). For the CFSE proliferation assay, the allogeneiclymphocytes were stained prior to the stimulation with DCs using CellTrace™ CFSE Cell Proliferation Kit (Thermo Scientific) according to the manufacturer's instructions. After 5 days, the cells were harvested and stained with a fixable live/dead stain (Vivid), fixed and permeabilized as previously described [38]. Next, the cells were stained with the following antibodies: CD3-PerCP-Cy5.5, CD4-PE-Cy7 (eBiosciences, San Diego, CA, USA), CD8-Alexa Fluor 700 (Exbio), and Ki-67-PE (Exbio). For the CFSE proliferation assay, the staining was performed without the Ki-67-PE antibody. To determine the expression of TIM-3 and PD-1, the 5-day cultures were stained as described [33]. Briefly, the cells were rinsed with PBS with 2 mM EDTA and stained with antibodies: CD8-Alexa Fluor 700 (Exbio), CD4-PE-Cy7, CD3-PerCP-Cy5.5 (eBiosciences, San Diego, CA, USA), Tim-3-PE, and PD-1-APC (BioLegend, San Diego, CA, USA). The cells were stained for 30–50 min at 4°C. The cells were rinsed and supplemented with 100 ng/ml DAPI (Thermo Scientific). Thereafter, the stained cells were analyzed by flow cytometry, and the acquired data were processed with the software as described above.

### 2.7. Lymphocyte Stimulation and Intracellular Cytokine Staining

Allogeneic lymphocytes were stimulated with DCs as in the Ki-67 proliferation assay with the exception that 4 days after the stimulation, the cells were supplemented with brefeldin A (BioLegend), and the cells were next cultured for an additional 18 hr. The cells were then fixed, permeabilized, and stained as for the Ki-67 assay with the exception that instead of the Ki-67-specific antibody were used the following antibodies: IFN*γ*-PE and TNF*α*-APC (Becton Dickinson).

### 2.8. Statistical Analysis

The means and SEM were calculated by GraphPad Prism 9 (GraphPad Software, La Jolla, CA, USA) using the indicated sample size (*n*). The statistical significance ( ^*∗*^*P* < 0.05,  ^*∗∗*^*P* < 0.01,  ^*∗∗∗*^*P* < 0.001,  ^*∗∗∗∗*^*P* < 0.0001) was determined by repeated measures (RM) one-way ANOVA with Tukey's post-test. Graphical images were produced using Biorender.com (agreement number: NX25ZTJPKE).

## 3. Results

### 3.1. The Supernatant Conditioned with Thapsigargin-Stimulated LAD2 MCs Induces DC Maturation

**Figure 1:**
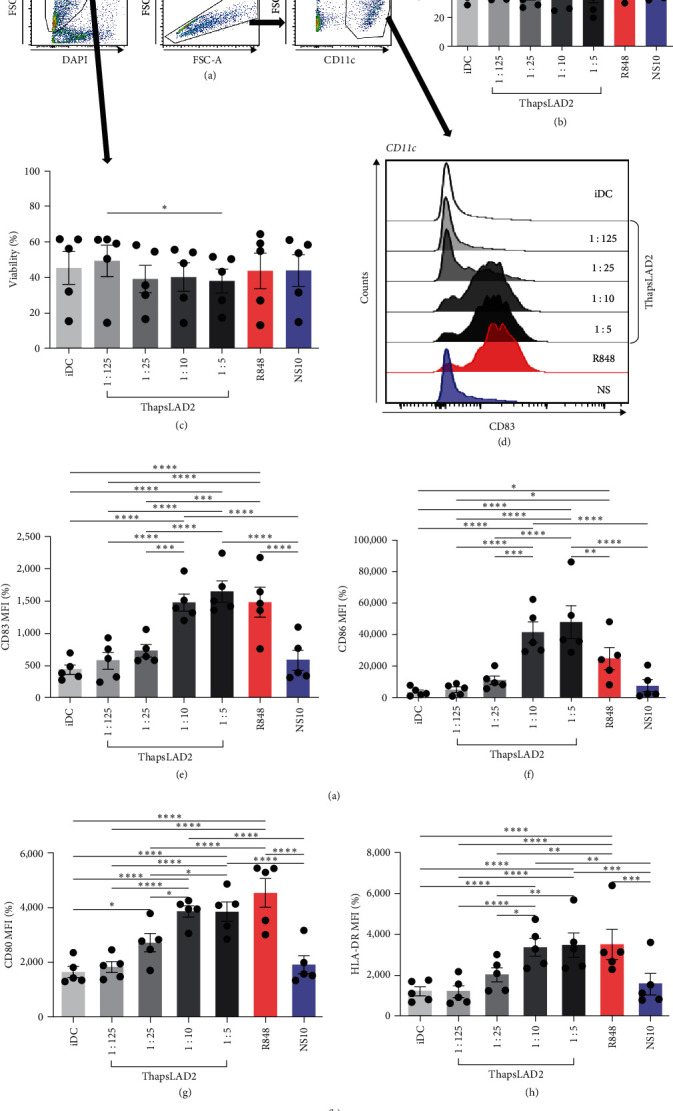
The supernatant conditioned with thapsigargin-stimulated LAD2 MCs induces DC maturation. (a) Flow cytometry data gating strategy. (b) The frequency of CD11c positive cells (DCs) in samples in (a) in iDC or iDC matured with 1 : 125-, 1 : 25-, 1 : 10-, or 1 : 5-diluted ThapsLAD2-conditioned medium (ThapsLAD2) or with R848 or 1 : 10-diluted VehicleLAD2-conditioned medium (NS10). (c) Cell viability of samples in (a). (d) A representative histogram of CD83 staining intensity. (e–h) The staining intensity of CD83 (e), CD86 (f), CD80 (g), and HLA-DR (h) in DCs in samples in (a). In (b), (c), and (e)–(h), bars represent the mean values and SEM determined in each group. The significance of differences among the group of cells is indicated ( ^*∗*^*P* < 0.05,  ^*∗∗*^*P* < 0.01,  ^*∗∗∗*^*P* < 0.001,  ^*∗∗∗∗*^*P* < 0.0001; *n* = 4 donors; RM one-way ANOVA with the Tukey post-test).

Our previous study has shown that a coculture of monocyte-derived DCs with thapsigargin-stimulated LAD2 MC line can induce the maturation of DCs [32]. Thapsigargin is an inhibitor of SERCA [25, 26, 27] and induces MC degranulation via activation of calcium signaling [29]. To learn whether the DC maturation was contact (coculture)-dependent or can be solely mediated via the biologically active products released after the thapsigargin-mediated stimulation, we stimulated LAD2 MCs with thapsigargin for 30 min, extensively rinsed them with culture medium, and then cultured them for 18–24 hr to condition the culture medium with the released products (thapsLAD2 medium). Thereafter, the thapsLAD2 medium was added to *ex vivo*-generated immature monocyte-derived DCs [32, 34], and the DCs were cultured for 18–24 hr. As shown, the thapsLAD2 medium did not compromise the viability or the content of DCs (Figures 1(a), 1(b), and 1(c)). However, the thapsLAD2 medium induced DC maturation as determined by the increased expression of DC maturation markers CD80, CD83, CD86, and HLA-DR (Figures 1(d), 1(e), 1(f), 1(g), and 1(h)). As shown, the extent of the marker expression was proportionally dependent on the dilution of the conditioned medium. The 10 and 5 times diluted thapsLAD2 medium already enhanced the expression of CD83, CD80, and HLA-DR as the TLR7/8-agonist, R848 [39], which was used as a positive control (Figures 1(e), 1(g), and 1(h)). The expression of CD86 was even significantly higher after the maturation with five times diluted thapsLAD2 medium than with R848 (Figure 1(f)). This indicated that thapsLAD2 medium can even more selectively enhance the expression of this costimulatory molecule, which is known to primarily support regulatory T-cell proliferation, survival, and maintenance in the presence of high levels of CTLA-4 inhibitory molecules [40] and whose expression is regulated by ubiquitinylation [41].

As further shown, no DC maturation was observed with the medium conditioned with resting LAD2 MCs (Figures 1(d), 1(e), 1(f), 1(g), and 1(h); NS10). These data show that DC maturation can be attained with thapsigargin-induced LAD2 MC released products without a DC coculture with LAD2 MCs and thus is not contact-dependent.

### 3.2. ThapsLAD2-Matured DCs Do Not Produce TNF*α* or IL-12

**Figure 2:**
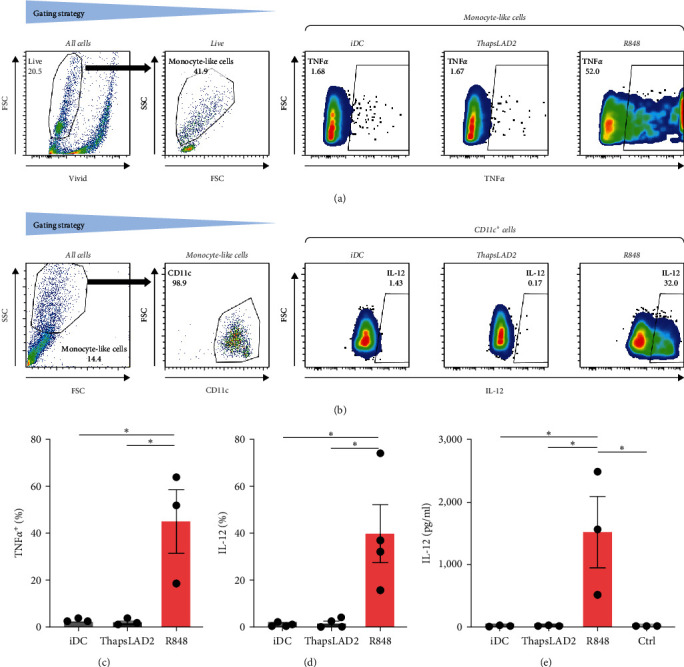
ThapsLAD2-matured DCs do not produce TNF*α* or IL-12. (a, b) Flow cytometry data gating strategy and a representative dot plot of TNF*α* (a) and IL-12 (b) intracellular staining in iDC or iDCs matured with ThapsLAD2 10-times diluted conditioned medium (ThapsLAD2) or with R848. (c, d) The frequency of TNF*α*- (c) or IL-12-producing (d) DCs in samples in (a) and (b). In (e), the calculated concentrations (pg/mL) of IL-12 released during DC maturation into the supernatants by 1 × 10^6^ cells/ml. The negative control was the sample without DCs (Ctrl). In (c)–(e), bars represent the mean values and SEM determined in each group. The significance of differences among the group of cells is indicated ( ^*∗*^*P* < 0.05; *n* = 3 (TNF*α*), 4 (IL-12), or 3 (released IL-12) donors; RM one-way ANOVA with the Tukey post-test).

The effective proinflammatory functionality of matured DCs depends on their ability to produce proinflammatory cytokines TNF*α* and IL-12 during their maturation [42]. We next investigated whether the DC maturation with thapsLAD2 medium also induced the production of TNF*α* and IL-12 in the maturing DCs. As shown, DC maturation with R848 significantly increased the frequency of TNF*α*- and IL-12-producing DCs as compared with immature DCs (Figure 2). However, the frequency of TNF*α*- and IL-12-producing DCs was minimal in the DCs maturing after the stimulation with thapsLAD2 medium (Figure 2). Next, we determined the extent of the cytokine released after DC maturation. Since TNF*α* is *de novo* produced after MC stimulation [43], and the thapsLAD2-conditioned medium is obtained after MC stimulation, which could thus interfere with the determination of TNF*α* produced by the maturing DCs, we analyzed only IL-12. As shown in Figure 2, the supernatant-released IL-12 was only detected in R848-matured DCs. No IL-12 was detected in the supernatant with iDCs nor thapsLAD2-matured DCs. These results, therefore, confirmed the findings of the flow cytometry analysis. Besides, since no detectable IL-12 was detected in the thapsLAD2 medium, the results also showed that thapsigargin-stimulated LAD2 MCs did not condition thapsLAD2 medium with *de novo* produced and released IL-12 (Figure 2(e); Ctrl). These findings show that although thapsLAD2 medium, comparably to R848, elevates the expression of DC maturation markers, it fails to induce the production of the key proinflammatory cytokines, TNF*α* and IL-12.

### 3.3. ThapsLAD2-Matured DCs Have Comparable but Increased Expression of, Respectively, PD-L1 and TIM-3, as Compared with Immature or R848-Matured DCs

**Figure 3:**
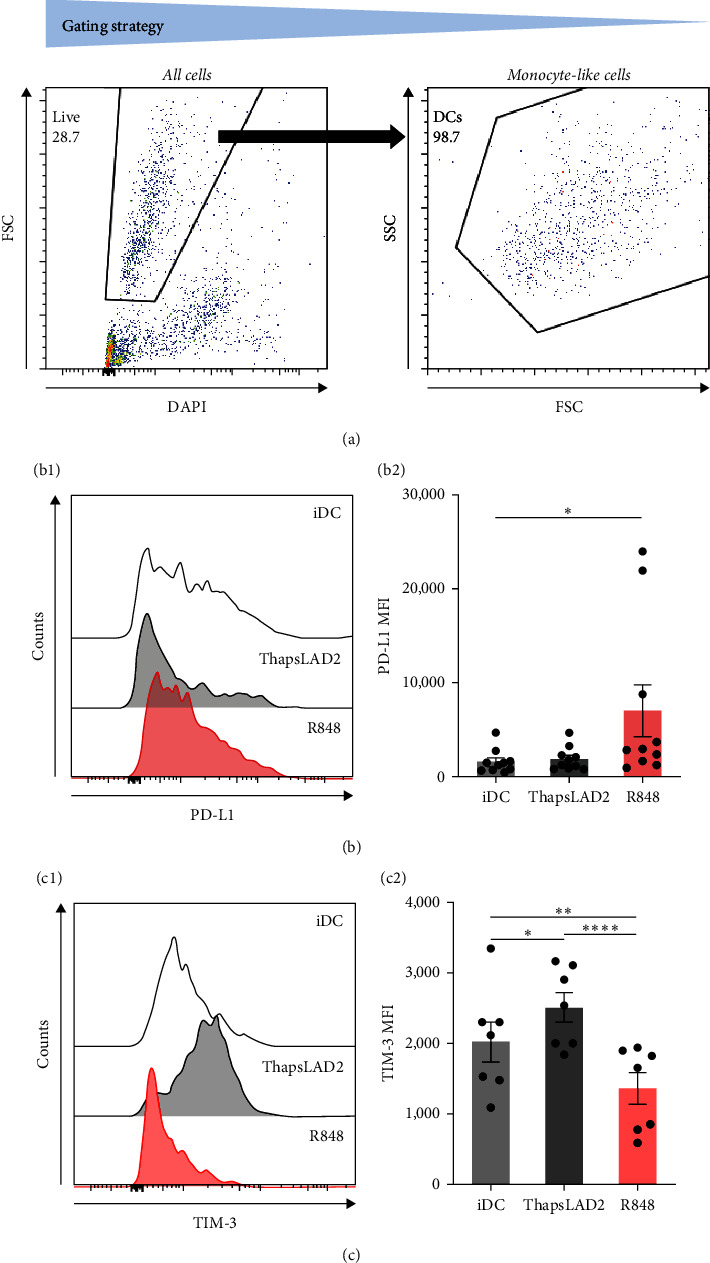
ThapsLAD2-matured DCs have comparable but increased expression of, respectively, PD-L1 and TIM-3, as compared with immature or R848-matured DCs. (a) Flow cytometry data gating strategy. (b) A representative histogram of PD-L1 staining intensity (b1) and its evaluation (b2) in iDCs (monocyte-like cells) or iDCs matured with ThapsLAD2 10-times diluted conditioned medium (ThapsLAD2) or with R848. (c) A representative histogram of TIM-3 staining intensity (c1) and its evaluation (c2) in iDCs (monocyte-like cells) or iDCs matured with ThapsLAD2 10-times diluted conditioned medium (ThapsLAD2) or with R848. In (b) and (c), bars represent the mean values and SEM determined in each group. The significance of differences among the group of cells is indicated ( ^*∗*^*P* < 0.05,  ^*∗∗*^*P* < 0.01,  ^*∗∗∗*^*P* < 0.001,  ^*∗∗∗∗*^*P* < 0.0001; *n* = 10 (b) or 7 (c) donors; RM one-way ANOVA with the Tukey post-test).

The data indicated that the phenotype of thapsLAD2-matured DCs could be different from the R848-matured DCs. One of the key molecules defining the functionality of DCs is the surface expression of two immune checkpoint molecules, PD-L1 and TIM-3 [44, 45]. Further investigation revealed comparable levels of PD-L1 expression in immature and thapsLAD2-matured DCs and a tendency of R848-matured DCs to display an increase in these levels (Figures 3(a) and 3(b)). However, thapsLAD2-matured DCs were found to significantly express more TIM-3 on their surface than immature DCs (Figure 3(c)). This level of TIM-3 expression contrasted with the level found in the R848-matured DCs, which was not only significantly lower than in the thapsLAD2-matured DCs but also in the immature DCs (Figure 3(c)). These data show that whereas the thapsLAD2 medium-mediated DC maturation enhances the surface expression of TIM-3 in the matured DCs, the opposite occurs after the DC maturation with R848.

### 3.4. ThapsLAD2-Matured DCs Have an Impaired Ability to Induce the Proliferation of CD8^+^ T Cells

**Figure 4:**
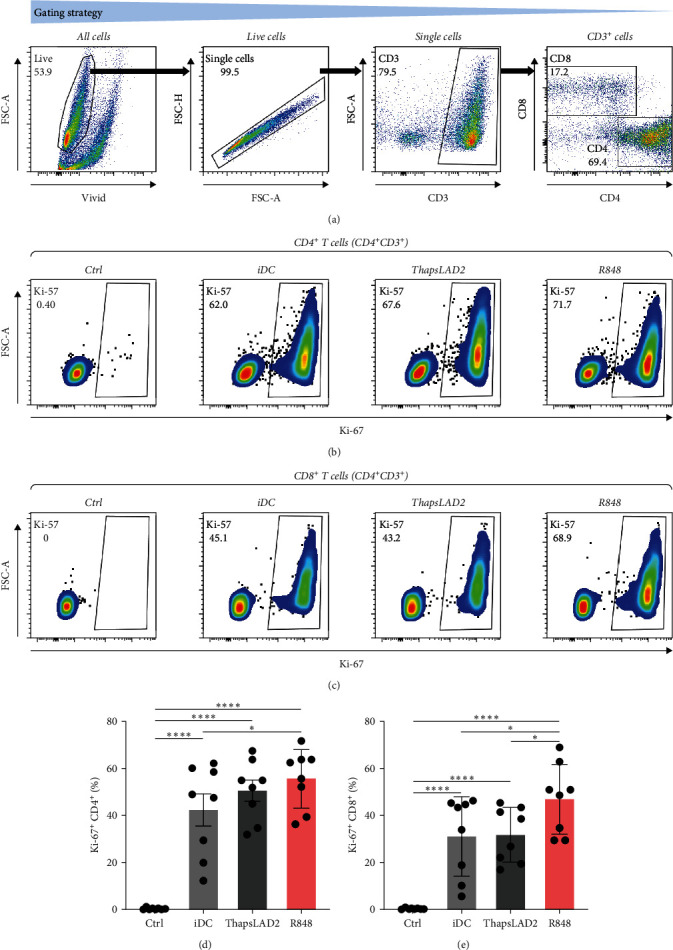
ThapsLAD2-matured DCs have an impaired ability to induce proliferation of CD8^+^ T cells as determined by Ki-67 expression. (a) Flow cytometry data gating strategy. (b, c) Representative dot plots of Ki-67 frequencies in CD4^+^ T cells (b) or CD8^+^ T cells (c) stimulated with iDCs or iDCs matured with ThapsLAD2 10-times diluted conditioned medium (ThapsLAD2) or with R848. The negative control was the sample stimulated with vehicle alone (Ctrl). (d, e) The frequencies of Ki-67-positive CD4^+^ T cells (d) or CD8^+^ T cells (e) in samples in (b) and (c), respectively. In (d) and (e), bars represent the mean values and SEM determined in each group. The significance of differences among the group of cells is indicated ( ^*∗*^*P* < 0.05,  ^*∗∗*^*P* < 0.01,  ^*∗∗∗*^*P* < 0.001,  ^*∗∗∗∗*^*P* < 0.0001; *n* = 8 donors; RM one-way ANOVA with the Tukey post-test).

**Figure 5:**
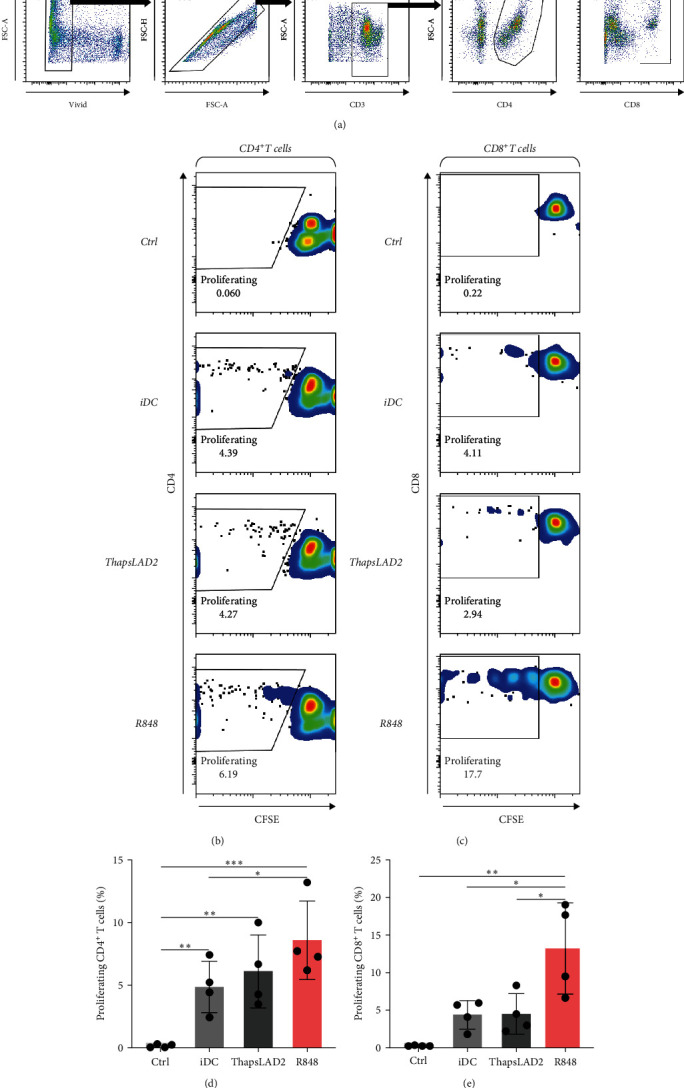
ThapsLAD2-matured DCs have an impaired ability to induce proliferation of CD8^+^ T cells as determined by CFSE proliferation assay. (a) Flow cytometry data gating strategy. (b, c) Representative dot plots of proliferating CFSE-stained CD4^+^ T cells (b) or CD8^+^ T cells (c) stimulated with iDCs or iDCs matured with ThapsLAD2 10-times diluted conditioned medium (ThapsLAD2) or with R848. The negative control was the sample stimulated with vehicle alone (Ctrl). (d, e) The frequencies of proliferating CFSE-stained CD4^+^ T cells (d) or CD8^+^ T cells (e) in samples in (b) and (c), respectively. In (d) and (e), bars represent the mean values and SEM determined in each group. The significance of differences among the group of cells is indicated ( ^*∗*^*P* < 0.05,  ^*∗∗*^*P* < 0.01,  ^*∗∗∗*^*P* < 0.001, *n* = 4 donors; RM one-way ANOVA with the Tukey post-test).

The crucial parameter of the functionality of matured DCs is their enhanced ability to induce the proliferation of T cells as compared with immature DCs [46, 47]. T-cell proliferation is significantly corroborated by TNF*α* and IL-12 [19, 48]. The failure of thapsLAD2-matured DCs to produce these cytokines during their maturation indicated that these DCs could also have a decreased ability to induce T-cell proliferation. To test this ability, we used allogeneic lymphocytes [49] and evaluated T cells after coculture with DCs using Ki-67 as a surrogate marker of their proliferation [50]. As shown, the frequency of proliferating Ki-67^+^ CD4^+^ T cells was significantly elevated in lymphocytes stimulated with R848-matured DCs as compared with lymphocytes stimulated with immature DCs (Figures 4(a), 4(b), and 4(d)). The samples stimulated with thapsLAD2-matured DCs showed only a tendency to the elevated frequency of Ki-67^+^ CD4^+^ T cells within the tested number of donors (Figure 4(d)). However, not even such a tendency was apparent for CD8^+^ T cells. As shown in Figure 4(c) and 4(e), both samples stimulated with immature or thapsLAD2-matured DCs showed comparable frequencies of proliferating Ki-67^+^ CD8^+^ T cells. On the other hand, the frequency of Ki-67^+^ CD8^+^ T cells was significantly elevated in samples stimulated with R848-matured DCs as compared with immature- or thapsLAD2-matured DCs-stimulated samples (Figure 4(e)). The findings of the Ki-67-based assay were also confirmed with the CFSE-based proliferation assay (Figure 5). These data show that whereas the DC maturation with R848 is projected into the increased ability of DCs to induce CD8^+^ T-cell proliferation, no such projection is found after DC maturation with thapsLAD2 medium.

### 3.5. ThapsLAD2-Matured DCs Induce T Cells with Comparable Expression of TIM-3 and PD-1 as Immature DCs

**Figure 6:**
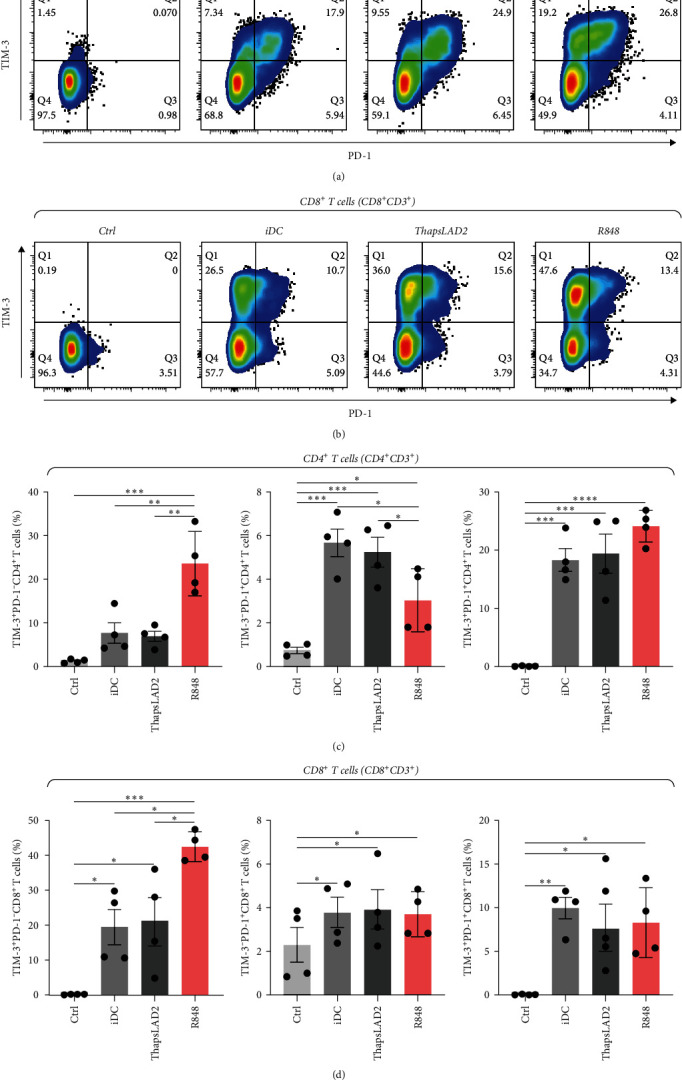
ThapsLAD2-matured DCs induce T cells with comparable expression of TIM-3 and PD-1 as immature DCs. (a, b) Representative dot plots of TIM-3 and/or PD-1 expression in CD4^+^ T cells (a) or CD8^+^ T cells (b) stimulated with iDCs or iDCs matured with ThapsLAD2 10-times diluted conditioned medium (ThapsLAD2) or with R848. The negative control was the sample stimulated with vehicle alone (Ctrl). (c, d) The frequencies of TIM-3- and/or PD-1-expressing CD4^+^ T cells (c) or CD8^+^ T cells (d) in samples in (a) and (b), respectively. In (c) and (d), bars represent the mean values and SEM determined in each group. The significance of differences among the group of cells is indicated ( ^*∗*^*P* < 0.05,  ^*∗∗*^*P* < 0.01,  ^*∗∗∗*^*P* < 0.001,  ^*∗∗∗∗*^*P* < 0.0001; *n* = 4 donors; RM one-way ANOVA with the Tukey post-test).

To further determine the functionality of ThapsLAD2-matured DCs, we have analyzed the expression of TIM-3 and PD-1 immune checkpoint molecules in allogeneic lymphocytes [49] after 5-day stimulation with DCs. As shown in Figure 6, these DCs induced the expression of both molecules on the surface of CD4^+^ and CD8^+^ T cells. No such expression was determined for T cells that were not stimulated with DCs. Different types of DC maturations had no impact on the frequencies of T cells expressing both checkpoint molecules. No such impact was also observed for CD8^+^ T cells expressing PD-1 without TIM-3 (TIM-3^−^ PD-1^+^). However, the CD4^+^ T cell counterpart stimulated with iDCs or ThapsLAD2-matured DCs showed comparable frequencies of PD-1-expressing TIM-3-negative cells (TIM-3^−^ PD-1^+^), and these frequencies were significantly higher than in T cells stimulated with R848-matured DCs. The most consistent differences among the groups were determined for PD-1-negative T cells expressing TIM-3 (TIM-3^+^PD-1^−^). As shown in Figure 6, T cells stimulated with iDCs or ThapsLAD2-matured DCs showed significantly much lower frequencies of TIM-3-expressing PD-1-negative T cells than T cells stimulated with R848-matured DCs. These data showed that ThapsLAD2-matured DCs produced T cells whose TIM-3/PD-1 expression profile was close to the one produced by iDCs and distant from the one produced by R848-matured DCs.

### 3.6. ThapsLAD2-Matured DCs Induce a Comparable Profile of IFN*γ*- and/or TNF*α*-Producing T Cells as Immature DCs

**Figure 7:**
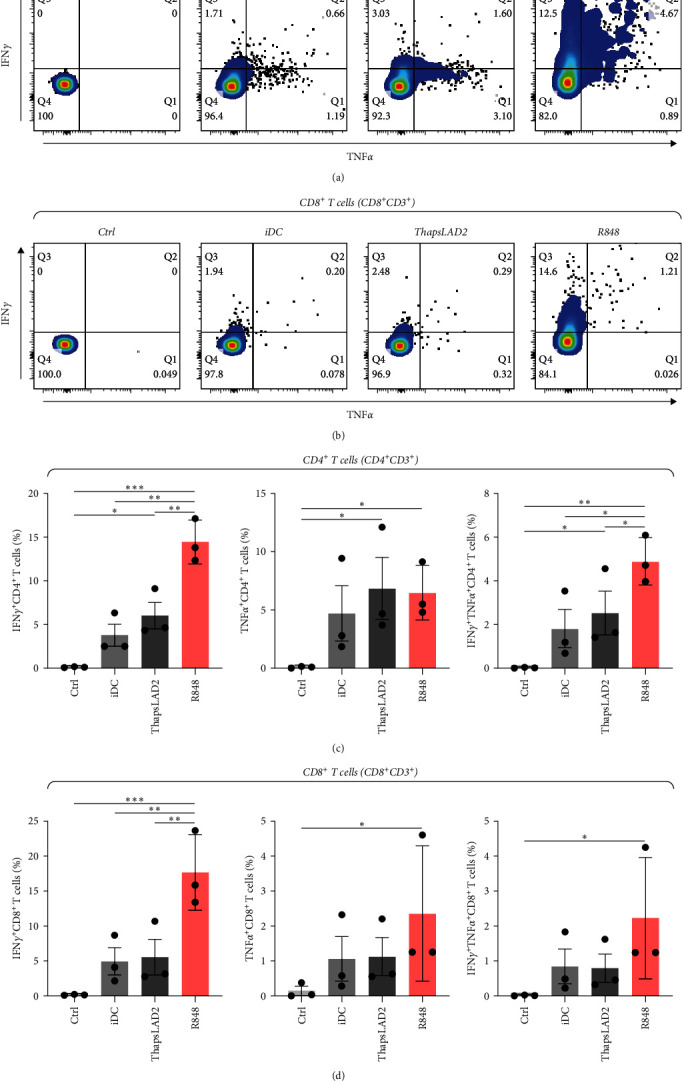
ThapsLAD2-matured DCs induce a comparable profile of IFN*γ*- and/or TNF*α*-producing T cells as immature DCs. (a, b) Representative dot plots of IFN*γ*- and/or TNF*α*-producing CD4^+^ T cells (a) or CD8^+^ T cells (b) stimulated with iDCs or iDCs matured with ThapsLAD2 10-times diluted conditioned medium (ThapsLAD2), or with R848. The negative control was the sample stimulated with vehicle alone (Ctrl). (c, d) The frequencies of IFN*γ*- and/or TNF*α*-producing CD4^+^ T cells (c) or CD8^+^ T cells (d) in samples in (a) and (b), respectively. In (c) and (d), bars represent the mean values and SEM determined in each group. The significance of differences among the group of cells is indicated ( ^*∗*^*P* < 0.05,  ^*∗∗*^*P* < 0.01,  ^*∗∗∗*^*P* < 0.001; *n* = 3 donors; RM one-way ANOVA with the Tukey post-test).

The functional differences between ThapsLAD2- and R848-matured DCs indicated the possibility of their translation also into functional differences of the DC-stimulated allogeneic T cells. Allogeneic lymphocytes were thus stimulated with DCs. After 4 days, the stimulated lymphocytes were supplemented with brefeldin A for 18 hr to allow intracellular accumulation of lymphocyte-produced cytokines. The lymphocytes were then analyzed for IFN*γ* and TNF*α*. As shown in Figure 7, the frequencies of TNF*α*-producing CD4^+^ T cells were comparable regardless of the type of induced DC maturation. However, a tendency to the enhanced frequencies of TNF*α*- and IFN*γ*/TNF*α*-producing CD8^+^ T cells was already shown when R848-matured DCs were used for lymphocyte stimulation. This tendency was already significant for IFN*γ*/TNF*α*-producing CD4^+^ T cells when much lower frequencies of the cytokines-producing cells were stimulated with iDC or ThapsLAD2-matured DCs than with R848-matured DCs. This significance was even much more evident for IFN*γ*-producing T cells, both CD4^+^ and CD8^+^ (Figure 7). These data showed that whereas R848-matured DCs had the capability to promote IFN*γ*- or IFN*γ*/TNF*α*-producing T cells, this capability was impaired in ThapsLAD2-matured DCs, which, despite their maturation, display a functionality comparable to iDCs.

## 4. Discussion

This study showed that monocyte-derived DCs could be efficiently matured with released products of thapsigargin-stimulated LAD2 MCs. However, this maturation was associated with increased surface expression of TIM-3 and diminished production of the key proinflammatory cytokines, TNF*α* and IL-12. In addition, although the maturation phenotype was associated with comparably enhanced expression of costimulatory molecules as in R848-matured DCs, their ability to induce T-cell proliferation and the generation of IFN*γ*- and IFN*γ*/TNF*α*-producing T cells was similar to iDCs.

**Figure 8:**
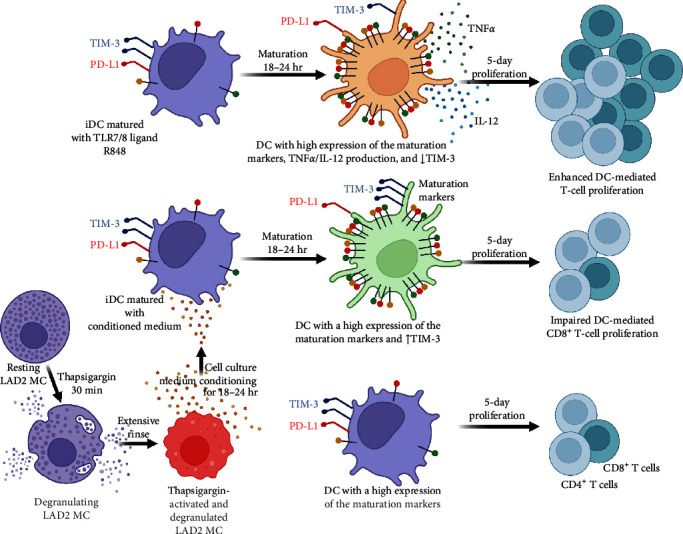
Schematic presentation of the findings of the study.

Many stimuli can activate MCs. In dependence on the type of stimulus, activated MCs selectively release biologically active products [51]. The release is mediated through regulated exocytosis and involves *de novo* synthesized or granule-prestored, presynthetized biologically active products [51, 52]. The release process occurs through different mechanisms [53]. Thapsigargin is a secretagogue that causes anaphylactic degranulation of MCs [29]. In LAD2 MCs, thapsigargin causes massive degranulation, during which over 80% of the granule content is released already after 30 min of stimulation [36]. This activation is also associated with *de novo* production of IL-8 and prostaglandin D2 (PGD2) [37], and presumably many other biologically active products yet to be fully determined in this MC cell line after the thapsigargin challenge. Regardless of which of these products or their combination contributed to DC maturation, these products seemed to be *de novo* synthesized since the granule-prestored products were found to be massively released from LAD2 MCs after the thapsigargin challenge [36] and were, under the experimental settings of this study, removed together with thapsigargin from the cell culture through extensive washing (Figure 8). In addition, following the extensive washing, these *de novo* products had to be released from the thapsigargin-stimulated LAD2 MCs during the subsequent 18–24 hr, during which the cells conditioned the culture medium (Figure 8).

DC maturation is a complex process during which DCs increase the surface expression of costimulatory or other molecules [42, 54]. The levels of their surface expression are considered hallmarks of DC maturation, and their levels [55, 56, 57] or stimulation-induced changes in their levels [58] in DCs, together with the DC numbers, are often considered prognostic markers in clinical specimens. Under many disease conditions, namely in tumors, the specimen infiltration with DCs with immature phenotype is often associated with immunosuppression or impaired immunostimulatory activities [59, 60, 61, 62, 63]. Indeed, immature DCs are often shown to have immunosuppressive phenotypes [64, 65]. However, the research has already accumulated evidence that also mature DCs can demonstrate immunosuppressive phenotype under certain conditions [66], including impaired ability to stimulate T-cell proliferation [67]. The findings of the present study even demonstrated one of these certain conditions *in vitro*, showing that *ex vivo*-produced immature DCs could be matured with the stimuli derived from thapsigargin-stimulated LAD2 MCs, but this stimuli-elicited maturation was not translated into an increased ability of DCs to induce T-cell proliferation, to promote IFN*γ*- or IFN*γ*/TNF*α*-producing T cells, or to generate a different TIM-3/PD-1 expression profile of the stimulated T cells than iDCs. These findings were largely contrasting with the parallel data showing that, under the same experimental conditions, the same immature DCs were matured with the TLR7/8-agonist R848 to the same extent, but this R848-elicited maturation was translated into the increased ability of DCs to induce T-cell proliferation, promote IFN*γ*- or IFN*γ*/TNF*α*-producing T cells, or to generate a different TIM-3/PD-1 expression profile of the stimulated T cells than iDCs. These findings, therefore, show that the DC maturation stimuli can comparably induce or enhance the expression of the cell surface DC maturation markers, many of which are costimulatory molecules known to enhance T-cell activation once ectopically overexpressed [68], but that this event does not necessarily need to translate into the promotion of the DC-mediated adaptive immune responses (Figure 8).

The functionality of matured DCs is also shaped by their production of inflammatory cytokines [42, 69]. R848 and other TLR agonists have been previously shown to induce DC maturation associated with the production of proinflammatory cytokines TNF*α* and IL-12 [70, 71]. TNF*α* is important for DC survival [72], their efficient maturation [73, 74, 75], and their potential to induce Th1 adaptive immune responses [75, 76]. TNF*α* seems to be also important for T-cell proliferation, and TNF-*α*-mediated signaling in T cells were found to be critically required for effective priming, proliferation, and recruitment of tumor-specific T cells [48].

The IL-12-producing mature DCs are highly immunogenic and also induce strong Th1 adaptive immune responses [77]. This materializes through a strong induction of sustained T-cell proliferation and differentiation of CD4^+^ T cells into Th1 cells and CD8^+^ T cells into efficient cytotoxic responses [71, 78, 79, 80]. IL-12 is often considered a third signal for CD8^+^ T-cell proliferation [19]. The data of the present study confirmed that R848-induced DC maturation was associated with enhanced TNF*α* and IL-12 production. However, these data contrasted with the finding that such enhancement was absent in the thapsLAD2-matured DCs. These results thus indicate that failure of thapsLAD2-matured DCs to augment enhanced proliferation of T cells may lie in the abrogated production of these cytokines [75, 80, 81, 82].

This study showed that thapsLAD2-matured DCs express higher levels of TIM-3 than immature DCs. TIM-3 expression on DCs is largely associated with their immunoregulatory or immunosuppressive phenotype [83]. Its mode of action in DCs is mediated via multiple mechanisms, including limiting the activation of the cGAS-STING pathway [84], which is necessary for cross-presentation of tumor-associated antigens [85, 86], mediating T-cell trogocytosis which labels these cells as a target for fratricide T-cell killing [87], or preventing increased exposure of intratumoral CD8^+^ T cells to DC-derived chemokines and cytokines, namely CXCL9 and IL-12 [88]. Many of these mechanisms can be limited either by using anti-TIM-3 blocking antibodies [84, 87, 88, 89] or forced downregulation of TIM-3 expression in DCs [90]. Whether these approaches before, during, or after the thapsLAD2-elicited maturation could modulate or alter the phenotype of the thapsLAD2-matured DCs remains to be investigated. However, regardless, this study showed that thapsLAD2-elicited maturation promotes TIM-3 expression, which contrasts with the TLR7/8-agonist-elicited maturation, in which the expression of TIM-3 in maturing DC decreases.

DCs are key players in adaptive antitumor immunity, and their ability to stimulate tumor-specific cytotoxic T cells is the critical mechanism through which the adaptive antitumor response materializes [91, 92]. However, the DC function is substantially controlled by the tumor microenvironment, which often reprograms DCs into cells with minimal antitumor activity [92, 93]. MCs are also present in the tumor microenvironment or at the periphery of tumors, contributing to the overall setting of the tumor microenvironment and its resistance to immunotherapy [94]. As both DCs and MCs possess strong immunomodulatory capabilities, they can act as cellular checkpoints in the tumor microenvironment, eliciting either pro- or antitumor activities [11]. This study showed how thapsigargin-activated MCs could, after their degranulation, produce biologically active compounds that have a distal impact on the key DC-mediated mechanisms of adaptive antitumor immune response. This impact materialized during the DC maturation and led to the impaired ability of DCs to induce T-cell proliferation and the generation of Th1 cytokines-producing T cells, which constitute the basis of Th1 antitumor immune responses [95]. The monocyte-derive DCs used in this study differ from other subsets of DCs [96] and can show dual pro- and anti-inflammatory features that can be elicited during their maturation [97]. We have previously shown that antimicrobial peptide LL-37 can modulate these DCs during differentiation and maturation, promoting their ability to stimulate CD8^+^ T cells [34]. On the other hand, the biologically active products of MCs, such as histamine, can reprogram maturing monocyte-derived DCs into DCs with anti-inflammatory properties [98]. Our study showed that these products could be *de novo* produced and secreted following thapsigargin-triggered LAD2 MC activation. However, regardless of which of these products were to mature and reprogram DCs into cells with impaired T-cell activation phenotype, these products are presumably associated with cellular signaling, the activation or inhibition of which could drive the DC reprograming during their maturation. One of these signaling recently identified was Wnt/*β*-catenin signaling [97]. Our study may indicate that other MC product-targeted signaling could be involved and, as such, become potential targets for interventions to increase the performance of not only tumor-infiltrating DCs *in vivo* but also monocyte-derived DCs produced *ex vivo* for active adoptive cellular immunotherapy of cancer [42].

## 5. Conclusion

This study demonstrates for the first time an *ex vivo* preparation of MC-matured monocyte-derived DCs with high expression of TIM-3, abrogated production of TNF*α* and IL-12, and impaired ability to induce proliferation of CD8^+^ T cells and the generation of IFN*γ*- and IFN*γ*/TNF*α*-producing T cells. This novel DC phenotype stresses the dichotomy between the DC maturation status and their functionality and thus corroborates the recent concept on the role of regulatory mature DCs in health and disease [66].

## Data Availability

The data used to support the findings of this study are available from the corresponding author upon request.
