# Corrigendum to “Impaired Proliferation of CD8^+^ T Cells Stimulated with Monocyte-Derived Dendritic Cells Previously Matured with Thapsigargin-Stimulated LAD2 Human Mast Cells”

**DOI:** 10.1155/jimr/9807058

**Published:** 2025-07-30

K. Kalkusova, P. Taborska, D. Stakheev, et al., “Impaired Proliferation of CD8^+^ T Cells Stimulated with Monocyte-Derived Dendritic Cells Previously Matured with Thapsigargin-Stimulated LAD2 Human Mast Cells,” *Journal of Immunology Research* 2024 (2024): 5537948, https://doi.org/10.1155/2024/5537948.

In the article, there are errors in Figure 1. The correct Figure 1 is shown below:

We apologize for this error.

## Figures and Tables

**Figure 1:**
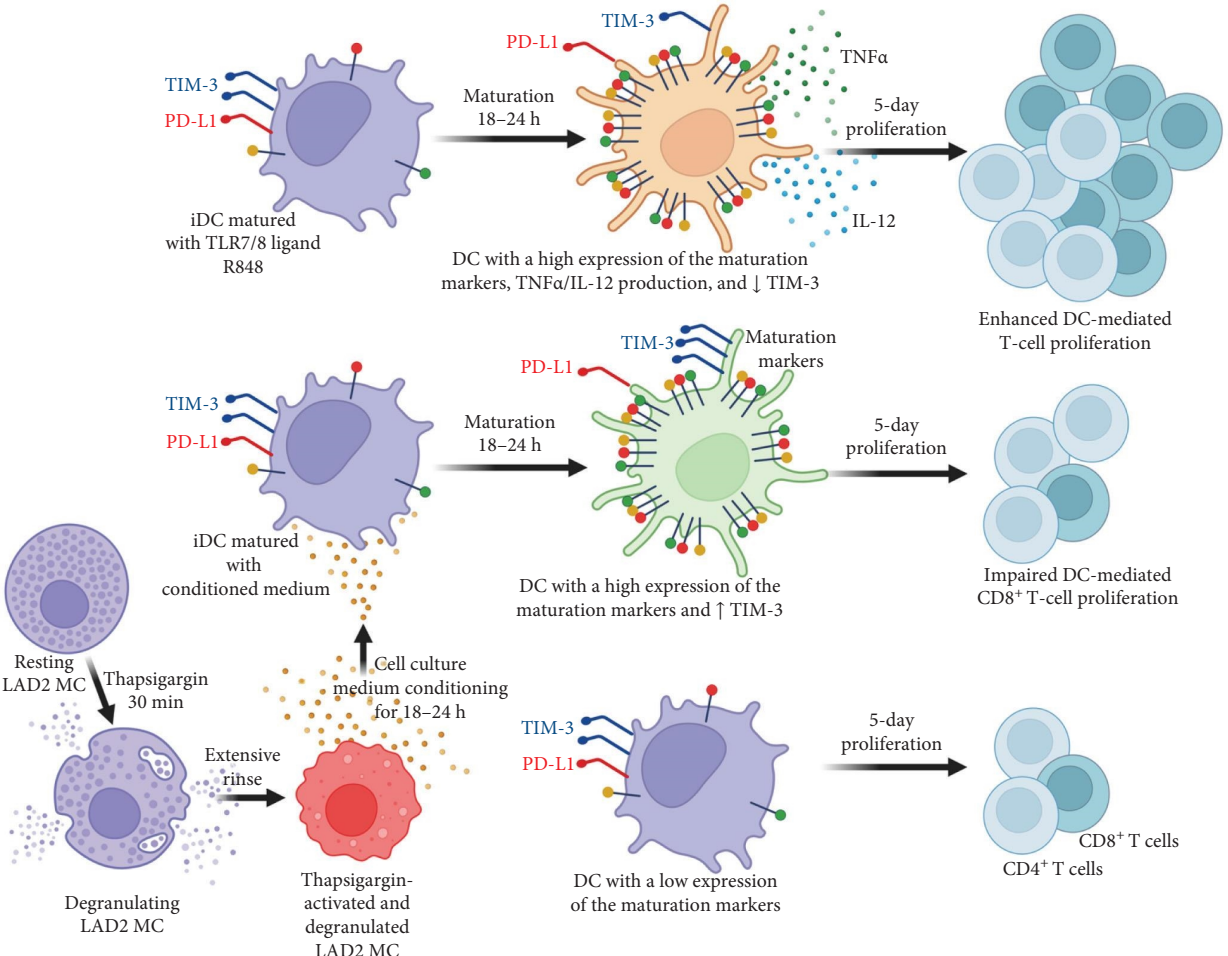
Schematic presentation of the findings of the study.

